# The role of the LncRNA XIST/miR-15a-5p/MN1 signaling axis in gender disparities in bladder cancer prognosis

**DOI:** 10.3389/fimmu.2025.1554829

**Published:** 2025-04-16

**Authors:** Fangzhen Cai, Siwei Xu, Yinan Li, Qingliu He, Qingfu Su, Heyi Chen, Weihui Liu, Jiabi Chen, Qingshui Wang, Yehuda G. Assaraf, Yao Lin, Wei Zhuang

**Affiliations:** ^1^ Department of Urology, The Second Affiliated Hospital of Fujian Medical University, Fujian Medical University, Quanzhou, Fujian, China; ^2^ The School of Clinical Medicine, Fuian Medical University, Fuzhou, China; ^3^ Department of Nephrology, The First Affiliated Hospital of Xiamen University, School of Medicine, Xiamen University, Xiamen, China; ^4^ Fujian-Macao Science and Technology Cooperation Base of Traditional Chinese Medicine-Oriented Chronic Disease Prevention and Treatment, Academy of Integrative Medicine, Fujian University of Traditional Chinese Medicine, Fuzhou, China; ^5^ The Fred Wyszkowski Cancer Research Laboratory, Faculty of Biology, Technion-Israel Institute of Technology, Haifa, Israel

**Keywords:** bladder cancer, gender disparity, XIST, MN1, Fzd2, targeted therapy

## Abstract

**Background:**

Bladder cancer (BC) exhibits significant gender disparities in incidence and prognosis, with women experiencing worse prognosis despite lower incidence rates. This study aims to elucidate the molecular mechanisms underlying these gender-specific differences, focusing on the role of the long non-coding RNA XIST.

**Methods:**

Comprehensive bioinformatics analysis was performed using TCGA and GSE13507 cohorts to identify gender-differential gene expression. Functional experiments including cell proliferation, migration, and invasion assays were conducted in bladder cancer cell lines. Molecular interactions were investigated through gene knockdown, overexpression, and luciferase reporter assays. A zebrafish model was employed to validate *in vivo* findings.

**Results:**

Our study revealed that XIST expression is significantly higher in female bladder cancer tissues and strongly associated with poor prognosis in female patients. The XIST/miR-15a-5p/MN1/FZD2 signaling axis was found to play a critical role in promoting bladder cancer progression. Specifically, XIST upregulates MN1 by sponging miR-15a-5p, which in turn enhances FZD2 expression. Functional experiments demonstrated that XIST knockdown significantly inhibited bladder cancer cell proliferation, migration, and invasion, effects which could be reversed by FZD2 overexpression.

**Conclusions:**

The XIST/miR-15a-5p/MN1 signaling axis plays a critical role in the gender disparity observed in bladder cancer prognosis, particularly in women. Targeting this pathway may offer new therapeutic strategies for improving outcomes in female BC patients.

## Introduction

Bladder cancer (BC) ranks among the top nine most common malignant tumors globally (1). Unlike other malignancies, BC exhibits a particularly pronounced sex disparity regarding both prevalence and patient prognosis. Men are nearly four times more likely to develop BC than women. However, a study using data from the Ohio Cancer Incidence Surveillance System (OCISS), which included over 47,000 BC patients diagnosed from 1996 to 2016, found that women with BC tend to have a poorer prognosis (2, 3). It is generally believed that women have a higher proportion of advanced-stage tumors and extra-vesical invasion, leading to worse prognosis (4–6). Similarly, a large population-based study in Sweden, which included 36,344 patients diagnosed with urothelial bladder cancer from 1997 to 2014, also showed that women have a higher mortality rate (7). This decreased survival for women was particularly pronounced in the first two years after diagnosis. Even when accounting for factors like tumor stage, women with BC still have worse long-term survival, indicating that other factors contribute to the observed gender disparity in BC (7).

Researchers have extensively investigated the reasons for this gender difference in BC prevalence and survival (8–11). Various hypotheses have been proposed, but the main factors remain unclear. One potential explanation is the hormonal changes women experience throughout their lives. While estrogen appears to suppress tumor formation, it may paradoxically encourage the progression of BC (12). In addition to hormones, sex chromosomes are also thought to play a role in BC risk. For example, women with Turner syndrome, which involves partial or complete loss of an X chromosome, have a higher BC risk, while men with Klinefelter syndrome, who have an extra X chromosome, tend to have a lower BC risk (13, 14). Current research into how sex chromosome gene expression affects the gender disparity in BC is limited, mainly focusing on lysine demethylase 6A (KDM6A). *KDM6A* is located on the X chromosome and functions as a tumor suppressor by demethylating H3K27 (15, 16). Seth Lerner and colleagues found that 26% of muscle-invasive bladder cancers have mutations that inactivate KDM6A, affecting gene expression and BC prognosis through changes in chromatin (15). Their study indicated that lower KDM6A expression is tied to BC progression in female mice and predicts poorer disease-free survival (15). Furthermore, extensive mutations in *KDM6A* were identified in urothelial bladder carcinoma, with about 70% resulting in loss of gene expression and demethylase activity (16). Transcriptome analysis found several disrupted pathways, particularly the PRC2/EZH2 pathway, in *KDM6A*-mutated urothelial bladder carcinoma.

In this study, we first compared gene expression profiles in BC between sexes, aiming to identify genes that could help reduce the sex disparity in BC. We identified the long non-coding RNA X-inactive specific transcript (XIST) on the X chromosome as a key player in this disparity. High levels of XIST mRNA were linked to poor overall survival in female BC patients, and functional assays showed that XIST promotes tumor cell proliferation, migration, and invasion in both in *in vitro* and *in vivo*. These findings offer new insights into the gender differences in BC recurrence and prognosis, potentially leading to targeted therapies focused on improving clinical outcomes for female BC patients.

## Methods

### Data collection

In this study, transcriptome RNA sequencing data and associated clinical information for bladder cancer patients were obtained from The Cancer Genome Atlas (TCGA) cohort. Additionally, gene expression profiles and clinical data were retrieved from the GSE13507 cohort in the Gene Expression Omnibus (GEO) database. The protocol for retrieving microarray gene expression data adhered to the methodology outlined in our previous research (17).

### Patients and specimens

A total of 100 surgical specimens from bladder cancer patients were collected from the Second Affiliated Hospital of Fujian Medical University, including 50 male and 50 female patients. Each case was pathologically confirmed by two independent pathologists. This study was approved by the Ethics Committee of the Second Affiliated Hospital of Fujian Medical University and adhered to the principles of the Declaration of Helsinki. Written informed consent was obtained from all patients. Specimen information was stored in the hospital database and used for research purposes.

### RNA extraction and RT-PCR

Total RNA was isolated from cells or tissues using TRIzol^®^ reagents. The levels of mRNA and lncRNA were quantified using reverse transcription followed by qPCR using the PrimeScript™ RT-qPCR kit (Takara, Japan). For miRNA level detection, reverse transcription was performed using the PrimeScript™ miRNA RT-qPCR kit (Sangon Biotech, Shanghai, China). RT-PCR experiments utilized a SYBR Green Kit (Vazyme, China). Glyceraldehyde-3-phosphate dehydrogenase (GAPDH) served as the internal control for mRNA and lncRNA, while U6 was used as the reference for miRNA. The relative expression levels of target genes were determined using the 2^-△△CT^ method.

### Luciferase reporter assay

The binding sites of XIST, miR-15a-5p, and the 3′-UTRs were identified using the LncACTdb3.0 database (http://www.bio-bigdata.net/LncACTdb). The sequence of miR-15a-5p (ACGACGA) was predicted to bind to the sequences of both XIST and the 3′-UTRs. Subsequently, we synthesized miR-15a-5p with mutated binding sites (miR-15a-5p-Mut). Following the manufacturer’s instructions, XIST and MN1 were co-transfected with either mimic-NC, miR-15a-5p mimic, or miR-15a-5p-Mut into T24 cells, a human transitional cell carcinoma of the bladder. Relative luciferase activity was measured using the Dual-GLO Luciferase Assay System (Promega Corporation). Firefly luciferase activity was normalized to Renilla luciferase activity.

### Western blot analysis

Cells were harvested and lysed using RIPA buffer containing protease inhibitors (Roche Ltd, Basel, Switzerland). Protein concentrations were measured with the Micro BCA Protein Assay Kit (Pierce Biotechnology). Proteins were then separated by 10% SDS-PAGE and transferred onto Amersham Protran nitrocellulose membranes (GE Healthcare Life Sciences, Fairfield, USA). The membranes were incubated overnight at 4°C with primary antibodies against target proteins, including anti-MN1 (1:500, 24697-1-AP, Proteintech) and anti-HA-tag (1:10,000, 66006-2-Ig, Proteintech), diluted in blocking solution. Protein detection and quantification were performed using the Odyssey^®^ CLx Infrared Imaging System (LI-COR Biosciences).

### Immunohistochemical staining

To assess MN1 protein levels, immunohistochemical staining was conducted on 100 bladder cancer tissue specimens and their adjacent normal tissues. Sections (3 μm thick) from paraffin-embedded samples were deparaffinized with dimethylbenzene and rehydrated. Antigen retrieval was performed by immersing the sections in 10 mM sodium citrate buffer at pH 6.0 and autoclaving at 121°C for 2 minutes. Endogenous peroxidase activity was blocked by incubating the sections in 3% hydrogen peroxide for 10 minutes at room temperature, followed by rinsing with PBS. To prevent non-specific binding, the sections were treated with 10% goat serum (ZhongShan Biotechnology, China) for 30 minutes. The sections were then incubated overnight at 4°C with an anti-MN1 antibody at a 1:100 dilution (Proteintech, China). After three PBS washes, the sections were incubated with an HRP-conjugated secondary antibody (Maixin, China) for 30 minutes at room temperature. The immunoreaction was visualized using a 3,3’-diaminobenzidine solution (Maixin, China), and counterstaining was performed with 20% hematoxylin, followed by dehydration.

### Evaluation of immunostaining intensity

MN1 positivity was identified by the presence of brownish-yellow granules in the cytoplasm or nucleus. The scoring criteria were as follows: the proportion of positive tumor cells: 0-4% = 0; 5-24% = 1; 25-49% = 2; 50-74% = 3; and 75-100% = 4. The staining intensity was scored as follows: no staining = 0; weak staining = 1; moderate staining = 2; and strong staining = 3. The final score was calculated as the product of these two values.

### Cell culture

The cell lines used in this study included the normal bladder epithelial cell line SV-HUC-1 and the bladder cancer lines RT4 and T24, all obtained from the American Type Culture Collection (ATCC). RT4 cells were cultured in DMEM, SV-HUC-1 cells in F-12K medium, and T24 cells in McCoy’s 5A medium. Each medium was supplemented with 10% fetal bovine serum and 1% penicillin-streptomycin. The cultures were maintained in a humidified atmosphere of 5% CO_2_ at 37°C.

### Cell proliferation assay

Cell proliferation was assessed using the Cell Counting Kit-8 (CCK-8) assay (Quanshijin, China). Monolayer cells were detached using trypsin, resuspended in growth medium, and adjusted to a cell density of 2 x 10^4^ cells/mL. These cell suspensions were then seeded into four 96-well plates at a density of 2 x 10^3^ cells/well and incubated at 37°C in a 5% CO_2_ atmosphere. At specific time points (24, 48, and 72 hours), 10 µL of CCK-8 solution was added to each well one hour before measuring absorbance. Absorbance was measured at 450 nm using a microplate reader (BioTek, ELx808, USA).

### Migration assay

RT4 and T24 cells were seeded in six-well plates containing 3 mL of growth medium at a density of 1 × 10^6^ cells per well. To minimize the impact on cell viability, the cultures were serum-starved. When the cells reached approximately 90% confluence, a scratch was made in the cell monolayer using a 10-µL pipette tip. Detached cells were washed away with PBS, and wound closure was observed using an inverted microscope at 0 and 48 hours. The migration area was quantified using ImageJ software.

### Invasion assay

Cell invasion was evaluated using Transwell inserts with polycarbonate membranes (Costar; Corning Inc.). Forty-eight hours post-transfection, cells (2 × 10^4^ per well) were placed in the upper chamber with 200 µL of serum-free medium. The upper chambers were then incubated for 24 hours in a 24-well plate containing 200 µL of complete growth medium with 10% fetal bovine serum in the lower chambers.

### Zebrafish xenograft assay

Zebrafish larvae were obtained from Fuzhou Bio-Service Biotechnology Co. Ltd. (Fuzhou, China). RT4 and T24 cells were stained with a 5 µM concentration of the red-fluorescent lipophilic dye CellTracker™ CM-DiI (C700, Invitrogen, CA, USA) and subsequently transplanted into zebrafish larvae using a microinjection technique. Approximately 200 red-fluorescent-labeled cells were injected into each larva, with each experimental group containing ten zebrafish larvae. To evaluate bladder cancer (BC) cell proliferation within the zebrafish model, fluorescent images were captured at 2 hours and 48 hours following xenotransplantation. Additionally, tail fluorescence was documented at 2 hours and 24 hours post-injection to observe and assess the formation of distant metastases.

### Power analysis

According to the power analysis performed using G*Power software, at least 3 Zebrafish per group were required to achieve statistically significant differences between groups (standard deviation = 0.93, α = 0.05, statistical power = 0.8). Our study with 10 Zebrafish per group satisfied this requirement. We did not perform power analysis for *in vitro* experiments but followed the common practice of performing *in vitro* experiments 3 times or more independently.

### Statistical analysis

Data analysis was conducted using Prism 6.0 software (GraphPad Software). Differences between means were evaluated using one-way ANOVA or Student’s t-test. Survival analysis was performed utilizing the Cox proportional hazards regression model and the Kaplan-Meier method, with statistical significance determined by the log-rank test. A p-value < 0.05 was considered statistically significant.

## Results

### Differential gene expression and prognostic implications in male and female bladder cancer patients

**Figure 1:**
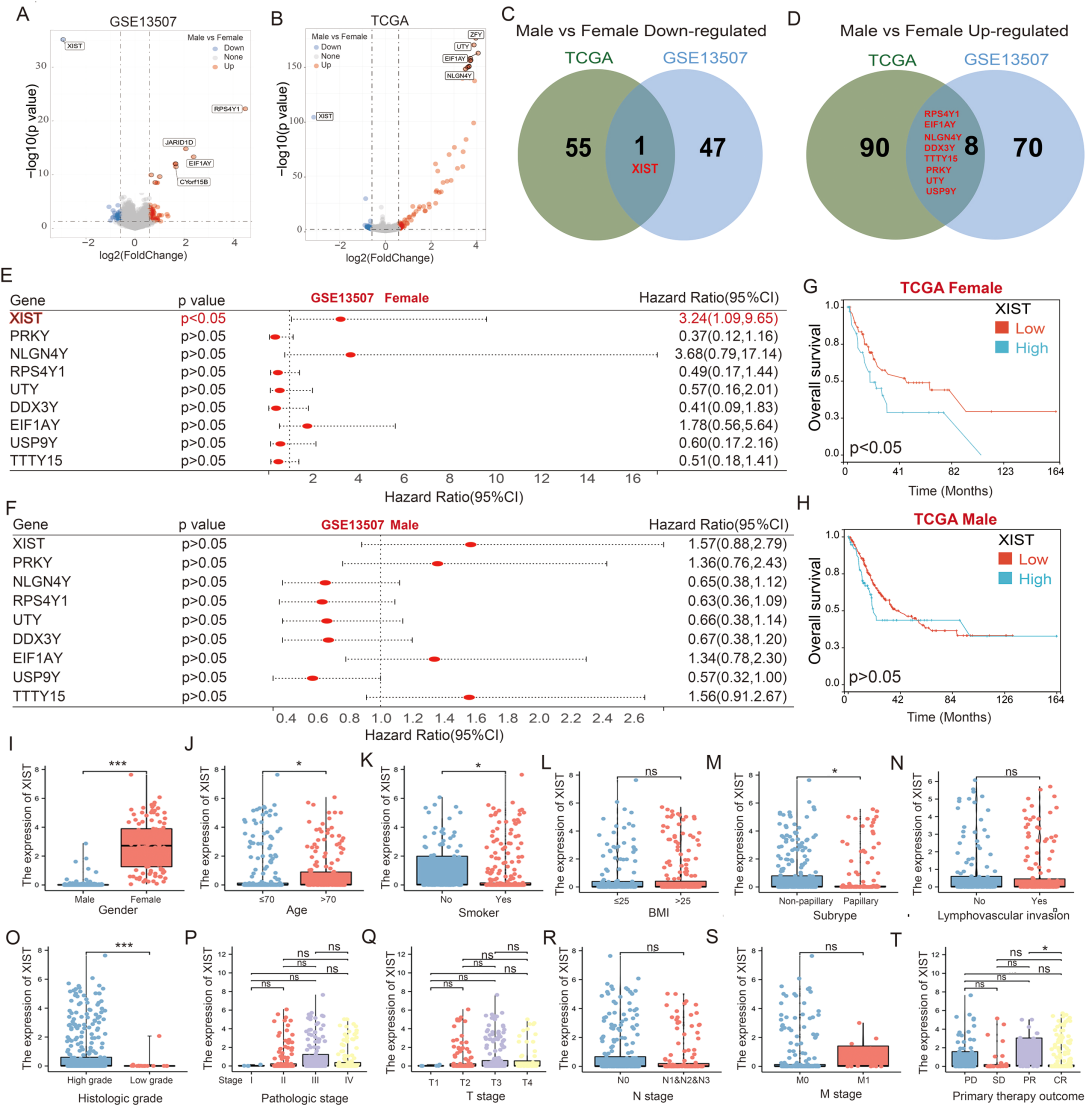
XIST is a critical gene associated with poor prognosis in female bladder cancer patients. **(A, B)** Volcano plots illustrating the differentially expressed genes between male and female bladder cancer patients in the **(A)** GSE13507 and **(B)** TCGA cohorts. **(C, D)** Venn diagrams showing the overlap of genes that are **(C)** downregulated and **(D)** upregulated in male versus female bladder cancer patients, as identified from the GSE13507 and TCGA datasets. **(E, F)** Prognostic significance of the intersecting genes analyzed using the GSE13507 cohort for **(E)** female and **(F)** male bladder cancer patients. **(G, H)** Impact of XIST expression on prognosis in **(G)** male and **(H)** female bladder cancer patients, evaluated using the TCGA cohort. **(I–T)** Expression analysis of XIST gene across various clinical subgroups in the TCGA cohort, including **(I)** gender, **(J)** age, **(K)** smoking history, **(L)** BMI, **(M)** tumor subtype, **(N)** lymphovascular invasion, **(O)** tumor grade, **(P)** tumor stage, **(Q)** T stage, **(R)** N stage, **(S)** metastatic stage, and **(T)** initial treatment outcome. ns, *p*>0.05; *p<0.05; ***p<0.001.

To investigate the differential genes that may be associated with prognosis in male and female bladder cancer patients, we initially analyzed the differentially expressed genes (DEGs) in tumor tissues from the GSE13507 cohort. Compared to male BC patients, 126 genes exhibited significantly altered expression in female BC patients, including 48 upregulated and 78 downregulated genes (**Figure 1A**). Additionally, we utilized the TCGA cohort to analyze DEGs in both male and female BC patients. Our analysis revealed that, compared to male BC patients, 154 genes had significantly altered expression in female BC patients, comprising 56 upregulated and 98 downregulated genes (**Figure 1B**). Venn diagram analysis indicated that XIST was the only gene consistently upregulated in female BC tissues across both the TCGA and GSE13507 cohorts (**Figure 1C**). In contrast, genes consistently upregulated in male BC tissues included neuroligin 4 Y-linked (NLGN4Y), ribosomal protein S4 Y-linked 1 (RPS4Y1), protein kinase Y-linked (PRKY), ubiquitously transcribed tetratricopeptide repeat containing Y-linked (UTY), DEAD-box helicase 3 Y-linked (DDX3Y), eukaryotic translation initiation factor 1A Y-linked (EIF1AY), testis-specific transcript Y-linked 15 (TTTY15), and ubiquitin-specific peptidase 9 Y-linked (USP9Y) (**Figure 1D**).

Next, we evaluated the prognostic implications of these nine genes in male and female BC patients using the GSE13507 cohort. Among female BC patients, high XIST expression was significantly associated with poorer overall survival compared to low XIST expression. The expression of the other genes did not significantly impact the prognosis of female BC patients (**Figure 1E**). In male BC patients, none of the nine genes significantly affected prognosis (**Figure 1F**). To corroborate these findings, we analyzed the impact of XIST expression on the prognosis of BC patients using the TCGA cohort. Consistent with the GSE13507 cohort, our results showed that female BC patients with high XIST expression had significantly poorer overall survival compared to those with low XIST expression (**Figure 1G**). XIST expression did not significantly influence the survival of male BC patients (**Figure 1H**). These findings suggest that increased expression of XIST in female BC tissues may contribute to the poorer prognosis observed in female BC patients.

Further analysis of the correlation between XIST expression and BC clinicopathological characteristics revealed significant correlations with gender (p < 0.001), age (p = 0.043), smoking status (p = 0.048), histological type (p = 0.028), cell differentiation (p < 0.001), and initial treatment outcome (p = 0.042). Conversely, XIST expression did not correlate with BMI (p = 0.988), lymphovascular invasion (p = 0.818), clinical stage (p = 0.237), T stage (p = 0.652), N stage (p = 0.291), or M stage (p = 0.929). Specifically, higher levels of XIST were observed in females, elderly patients, non-papillary BC cases, and high-grade tumors. Patients showing partial remission after initial treatment had higher XIST levels compared to those with complete remission. Additionally, smokers exhibited lower XIST levels compared to non-smokers (**Figures 1I–T**).

### XIST promotes proliferation and metastatic ability of bladder cancer cells *in vitro* and *in vivo*

**Figure 2:**
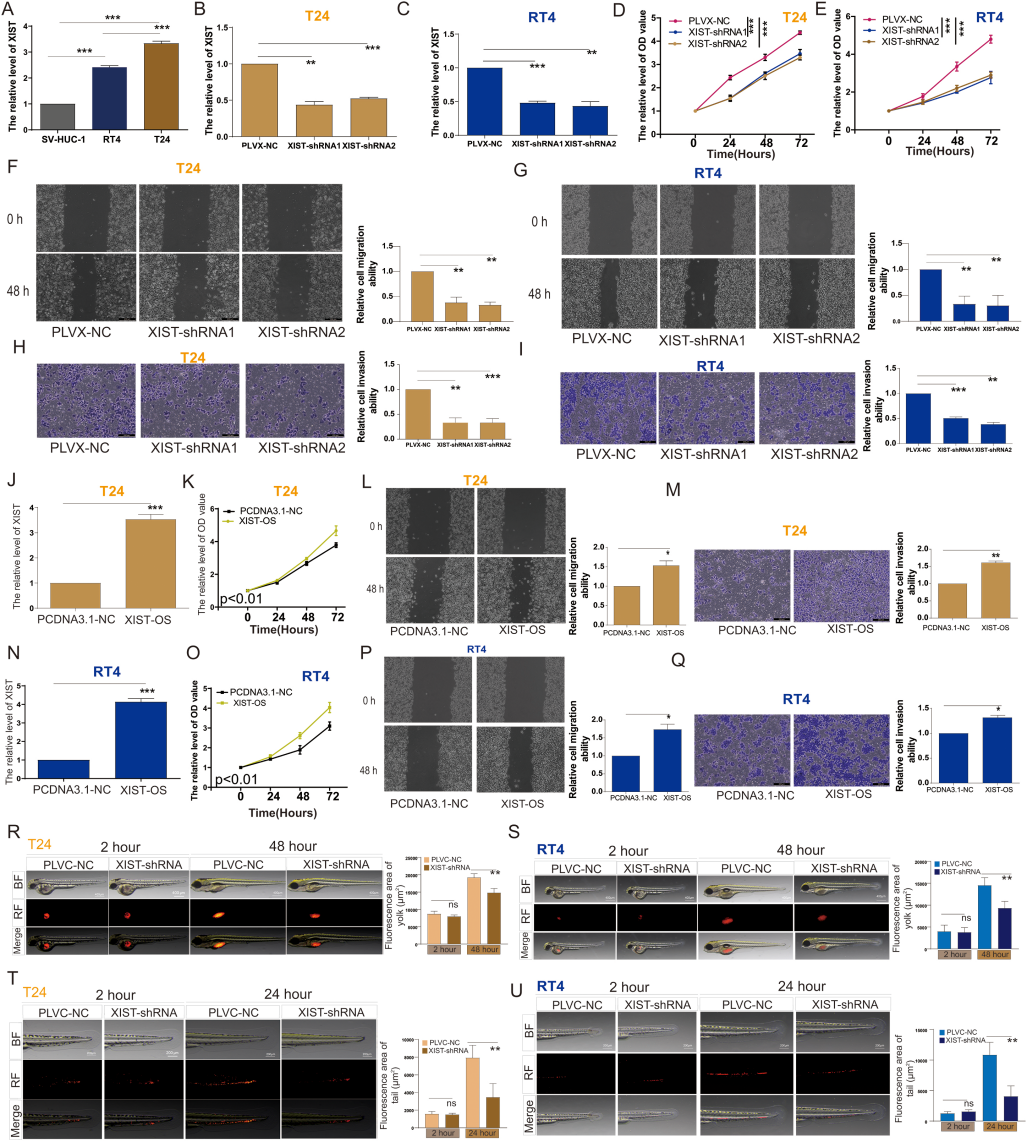
XIST promotes the proliferation and metastasis of bladder cancer cells both *in vitro* and *in vivo*. **(A)** Expression levels of XIST in SV-HUC-1, RT4, and T24 cells. **(B, C)** RT-PCR analysis of XIST expression following knockdown in **(B)** T24 and **(C)** RT4 cells. **(D, E)** Impact of XIST knockdown on the proliferation of **(D)** T24 and **(E)** RT4 cells. **(F, G)** Impact of XIST knockdown on the migration of **(F)** T24 and **(G)** RT4 cells. **(H, I)** Impact of XIST knockdown on the invasion of **(H)** T24 and **(I)** RT4 cells. **(J)** RT-PCR analysis of XIST expression following its overexpression in T24 cells. **(K)** Effect of XIST overexpression on the proliferation of T24 cells. **(L)** Effect of XIST overexpression on the migration of T24 cells. **(M)** Effect of XIST overexpression on the invasion of T24 cells. **(N)** RT-PCR analysis of XIST expression following its overexpression in RT4 cells. **(O)** Effect of XIST overexpression on the proliferation of RT4 cells. **(P)** Effect of XIST overexpression on the migration of RT4 cells. **(Q)** Effect of XIST overexpression on the invasion of RT4 cells. **(R, S)** Impact of XIST knockdown on the proliferation of **(R)** T24 and **(S)** RT4 cells in the zebrafish model. **(T, U)** Impact of XIST knockdown on the migration of **(T)** T24 and **(U)** RT4 cells in the zebrafish model. *p<0.05, **p<0.01, ***p<0.001. ns, p>0.05.

To evaluate the expression of the XIST gene in bladder cancer (BC) cells, we assessed XIST expression in SV-HUC-1 (normal bladder epithelial cells), RT4 (male BC-derived), and T24 (female BC-derived) cells. Our findings revealed that mRNA expression of XIST was significantly elevated in both RT4 and T24 cells compared to SV-HUC-1, with T24 cells exhibiting markedly higher expression than RT4 cells (**Figure 2A**). Subsequently, we established stable RT4 and T24 cell lines with either PLVC-NC (control group) or XIST knockdown using XIST-shRNA1 and XIST-shRNA2 (**Figures 2B, C**). Functional assays demonstrated that XIST knockdown significantly suppressed the proliferation, migration, and invasion capacities of both RT4 and T24 cell lines (**Figures 2D–I**). Conversely, overexpression of XIST markedly enhanced these cellular functions in both cell lines (**Figures 2J–Q**).

To explore the biological function of the XIST gene *in vivo*, we utilized a zebrafish tumor xenograft model to evaluate the impact of XIST on the proliferation and migration abilities of RT4 and T24 cells within a living organism. Zebrafish are an established vertebrate model that possesses signal transduction pathways fundamentally similar to those in humans, with their biological structures and physiological functions being highly conserved when compared to mammals. The inherent transparency of zebrafish allows for direct observation, making them an ideal model organism. Our zebrafish tumor model experiments clearly demonstrated that XIST knockdown significantly attenuated the proliferation and metastatic capabilities of RT4 and T24 cells *in vivo* (**Figures 2R–U**).

### Gender-specific implications of the XIST/miR-15a-5p/MN1 regulatory network in bladder cancer

**Figure 3:**
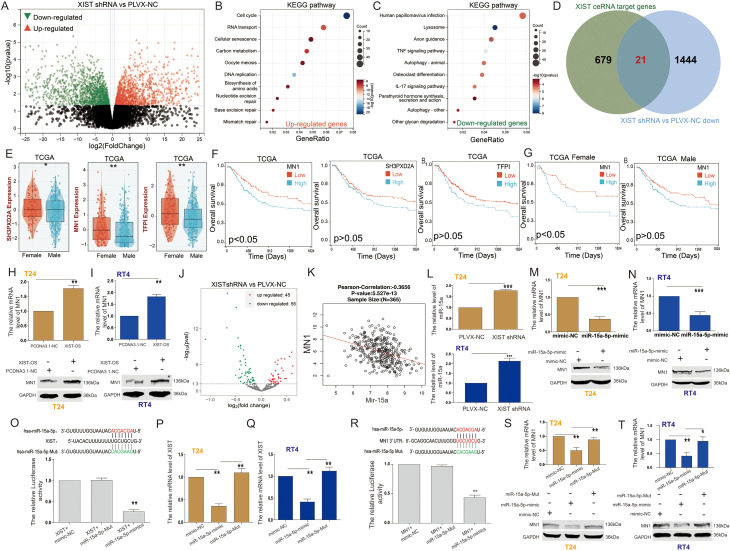
XIST regulates MN1 expression through miR-15a-5p. **(A)** Volcano plot illustrating the differentially expressed genes between XIST-shRNA and control groups in T24 cells. **(B, C)** KEGG pathway analysis of **(B)** upregulated and **(C)** downregulated genes following XIST knockdown. **(D)** Venn diagram showing the overlap between XIST ceRNA target genes and downregulated genes after XIST knockdown. **(E)** Expression levels of MN1, SH3PXD2A, and TFP1 genes in male and female bladder cancer patients. **(F)** Prognostic significance of MN1, SH3PXD2A, and TFP1 gene expression in bladder cancer patients. **(G)** Prognostic impact of MN1 expression in male and female bladder cancer patients. **(H)** Effect of XIST overexpression on MN1 mRNA and protein levels in T24 cells. **(I)** Effect of XIST overexpression on MN1 mRNA and protein levels in RT4 cells. **(J)** Volcano plot illustrating the differentially expressed miRNAs between XIST-shRNA and control groups in T24 cells. **(K)** Correlation analysis between MN1 and miR-15a expression. **(L)** Effect of XIST knockdown on miR-15a-5p expression in T24 and RT4 cells. **(M, N)** Effect of miR-15a-5p overexpression on MN1 mRNA and protein levels in **(M)** T24 and **(N)** RT4 cells. **(O)** Dual-luciferase reporter assay demonstrating the interaction between XIST and miR-15a-5p. **(P, Q)** Effect of miR-15a-5p on XIST expression in **(P)** T24 and **(Q)** RT4 cells. **(R)** Dual-luciferase reporter assay demonstrating the interaction between MN1 and miR-15a-5p. **(S, T)** Effect of miR-15a-5p on MN1 expression in **(S)** T24 and **(T)** RT4 cells. *p<0.05, **p<0.01, ***p<0.001.

To further elucidate the molecular mechanisms by which the XIST gene influences bladder cancer cell functions, we performed RNA sequencing analysis on T24 cells. The results revealed that, compared to the control PLVX-NC group, a total of 1,313 genes were significantly upregulated, while 1,465 genes were significantly downregulated in the XIST-shRNA group (**Figure 3A**). KEGG enrichment analysis indicated that the upregulated genes were mainly involved in pathways such as DNA replication, RNA transport, the cell cycle, and carbon metabolism (**Figure 3B**). Conversely, the downregulated genes were predominantly associated with pathways related to lysosomes, human papillomavirus infection, osteoclast differentiation, and autophagy (**Figure 3C**).

Using the LncACTdb3.0 database, we identified potential competing endogenous RNA (ceRNA) network genes regulated by XIST. Our analysis uncovered 700 potential target genes of XIST. By intersecting these genes with the 1,465 downregulated genes following XIST knockdown, we identified 21 genes that are both potential targets of XIST and positively regulated by it (**Figure 3D**). These 21 genes included CBX7, CD44, CDKN1A, DAB2, MN1, NTN4, PKD1, SALL4, SMAD7, SH3PXD2A, SYNGAP1, RIPK4, MAFG, STC2, NUMB, CDKN2B, PTGS2, WNT5A, TFPI, EREG, and RAB5B.

Next, we assessed the expression of these 21 genes in male and female BC patients using the TCGA cohort. The results indicated that only MN1, SH3PXD2A, and TFPI exhibited expression patterns similar to XIST, with significantly higher expression levels in the tissues of female BC patients compared to their male counterparts (**Figure 3E**). Survival analysis revealed that high MN1 expression serves as a prognostic factor associated with poor outcomes in BC patients (**Figure 3F**). Further analysis of the relationship between MN1 expression and survival in male and female BC patients showed results comparable to those of XIST. Elevated MN1 expression was linked to poor prognosis in female BC patients, while its expression had no significant impact on the prognosis of male BC patients (**Figure 3G**). Overexpression of XIST significantly increased the mRNA and protein levels of MN1 in both T24 cells (**Figure 3H**) and RT4 cells (**Figure 3I**).

Additionally, transcriptomic data analysis following XIST knockdown revealed changes in miRNA expression, with 45 miRNAs significantly upregulated and 55 miRNAs significantly downregulated (**Figure 3J**). Using the LncACTdb3.0 database, we identified that XIST, miR-15a-5p, and MN1 can form a ceRNA network. Correlation analysis through the LinkedOmics cohort indicated a significant negative correlation between miR-15a and MN1 expression in BC tissues (**Figure 3K**). Notably, XIST knockdown significantly increased miR-15a-5p expression in both T24 and RT4 cells (**Figure 3L**). Moreover, overexpression of miR-15a-5p significantly inhibited MN1 mRNA and protein levels in T24 cells (**Figure 3M**) and RT4 cells (**Figure 3N**).

To further explore the interaction between XIST and miR-15a-5p, we predicted the putative binding sites for miR-15a-5p on XIST mRNA using the LncACTdb3.0 analysis platform. The binding sites were identified as UGCUGCU and AGCAGCA. Dual-luciferase reporter assays demonstrated that miR-15a-5p significantly decreased the luciferase activity of XIST (**Figure 3O**). Furthermore, miR-15a-5p reduced the expression of the XIST gene in both T24 and RT4 cells (**Figures 3P, Q**). Additionally, we analyzed the binding sites between the MN1 3’ UTR and miR-15a-5p, confirming the same sites, UGCUGCU and AGCAGCA, respectively. Dual-luciferase reporter assays confirmed that miR-15a-5p significantly decreased the luciferase activity of the MN1 3’ UTR (**Figure 3R**). Furthermore, miR-15a-5p decreased MN1 expression in both T24 and RT4 cells (**Figures 3S, T**).

### XIST promotes bladder cancer cell proliferation and metastasis through the miR-15a-5p/MN1 pathway

**Figure 4:**
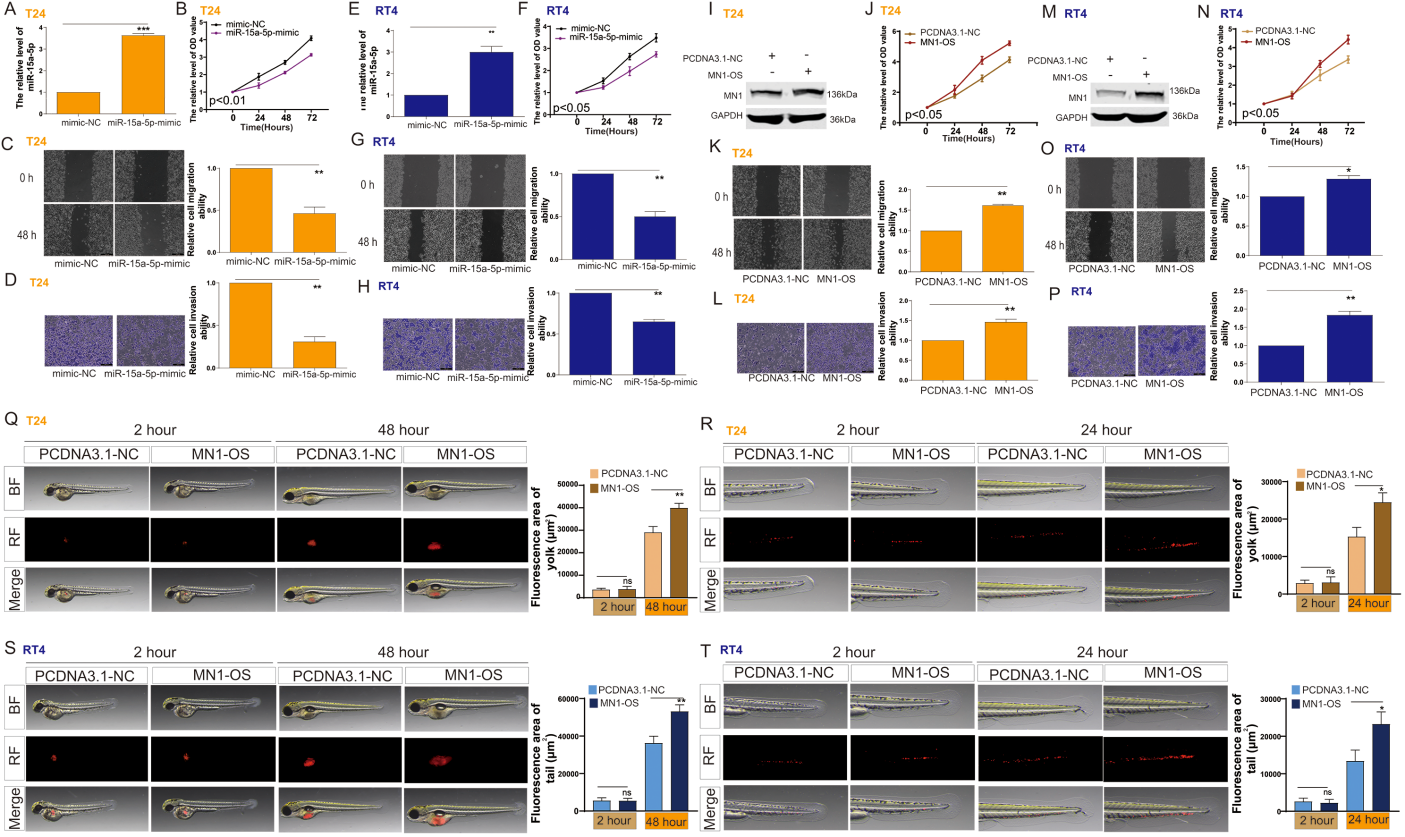
The impact of miR-15a-5p and MN1 on the proliferation and metastasis of bladder cancer cells. **(A)** RT-PCR analysis of miR-15a-5p expression in T24 cells following transfection with miR-15a-5p-mimic. **(B–D)** Impact of miR-15a-5p transfection on the **(B)** proliferation, **(C)** migration, and **(D)** invasion capabilities of T24 cells. **(E)** RT-PCR analysis of miR-15a-5p expression in RT4 cells following transfection with miR-15a-5p-mimic. **(F–H)** Impact of miR-15a-5p transfection on the **(F)** proliferation, **(G)** migration, and **(H)** invasion capabilities of RT4 cells. **(I)** Western blot analysis of MN1 expression in T24 cells following MN1 overexpression. **(J–L)** Impact of MN1 overexpression on the **(J)** proliferation, **(K)** migration, and **(L)** invasion capabilities of T24 cells. **(M)** Western blot analysis of MN1 expression in RT4 cells following MN1 overexpression. **(N–P)** Impact of MN1 overexpression on the **(N)** proliferation, **(O)** migration, and **(P)** invasion capabilities of RT4 cells. **(Q, R)** Impact of MN1 overexpression on the **(Q)** proliferation and **(R)** metastasis capabilities of T24 cells in the zebrafish model. **(S, T)** Impact of MN1 overexpression on the **(S)** proliferation and **(T)** metastasis capabilities of RT4 cells in the zebrafish model. *p<0.05, **p<0.01, ***p<0.001. ns, p>0.05.

**Figure 5:**
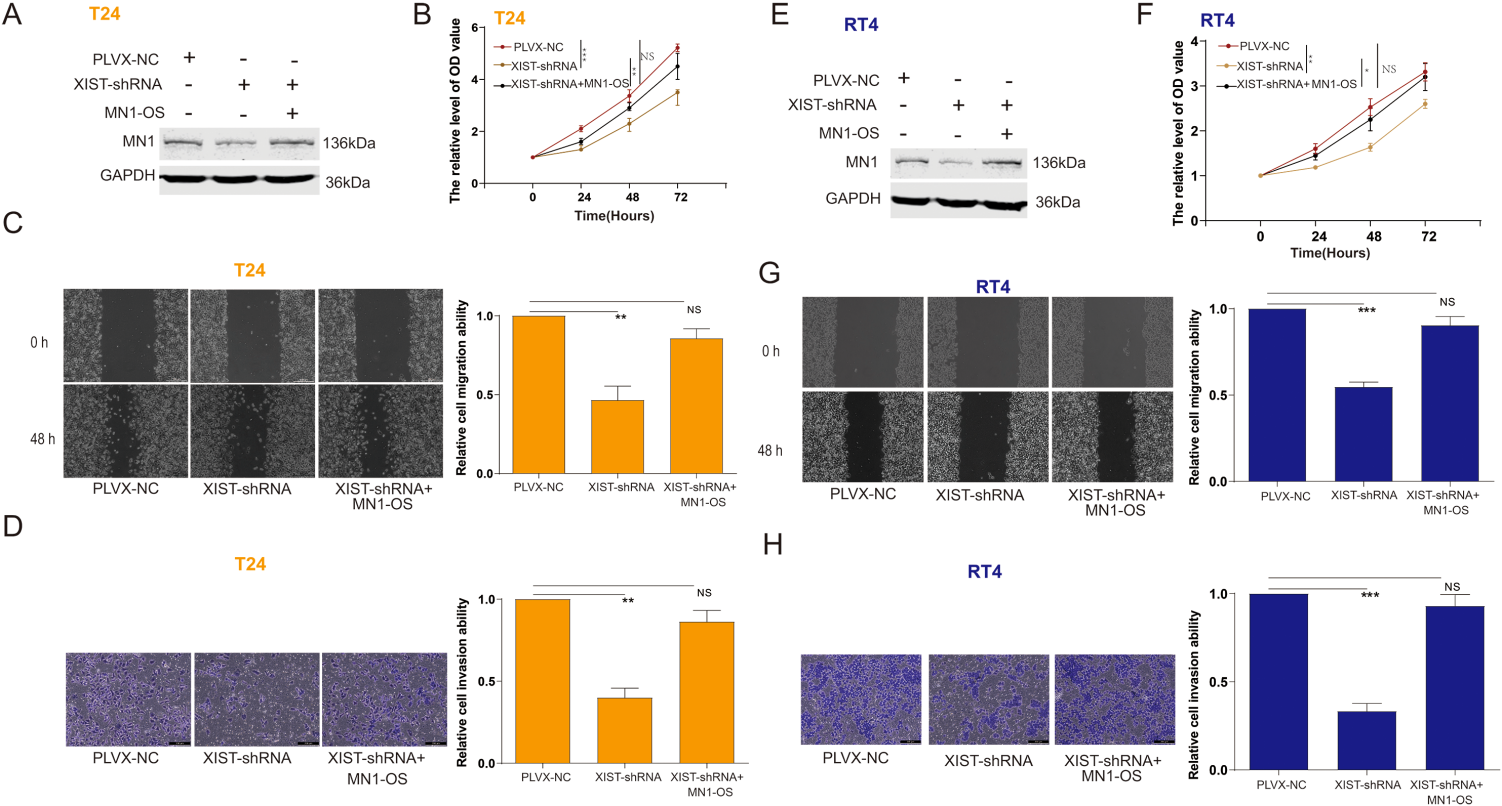
The impact of XIST on the proliferation and metastasis of bladder cancer cells is related to MN1. **(A)** Western blot analysis of MN1 expression in T24 cells after transfection with XIST-shRNA and MN1-OS. **(B–D)** Impact of XIST-shRNA and MN1-OS transfection on the **(B)** proliferation, **(C)** migration, and **(D)** invasion capabilities of T24 cells. **(E)** Western blot analysis of MN1 expression in RT4 cells after transfection with XIST-shRNA and MN1-OS. **(F–H)** Impact of XIST-shRNA and MN1-OS transfection on the **(F)** proliferation, **(G)** migration, and **(H)** invasion capabilities of RT4 cells. *p<0.05, **p<0.01, ***p<0.001. ns, p>0.05.

We investigated the effects of miR-15a-5p and MN1 on the functional capabilities of bladder cancer cells. The results indicated that overexpression of miR-15a-5p significantly inhibited the proliferation, migration, and invasion of T24 cells (**Figures 4A–D**) and RT4 cells (**Figures 4E–H**). In contrast, overexpression of MN1 significantly enhanced these capabilities in both T24 (**Figures 4I–L**) and RT4 cells (**Figures 4M-P**). Additionally, experiments using a zebrafish model demonstrated that overexpression of MN1 notably increased the proliferation and metastatic potential of both T24 (**Figures 4Q, R**) and RT4 (**Figures 4S, T**) cells *in vivo*. Furthermore, we examined whether the impact of XIST on bladder cancer cell functions is mediated by MN1. Our results revealed that knockdown of XIST significantly suppressed cell proliferation, migration, and invasion in T24 (**Figures 5A–D**) and RT4 cells (**Figures 5E–H**). Remarkably, these inhibitory effects were reversed by overexpression of MNl (**Figure 5**).

### Expression of XIST/miR-15a-5p/MN1 in bladder cancer

**Figure 6:**
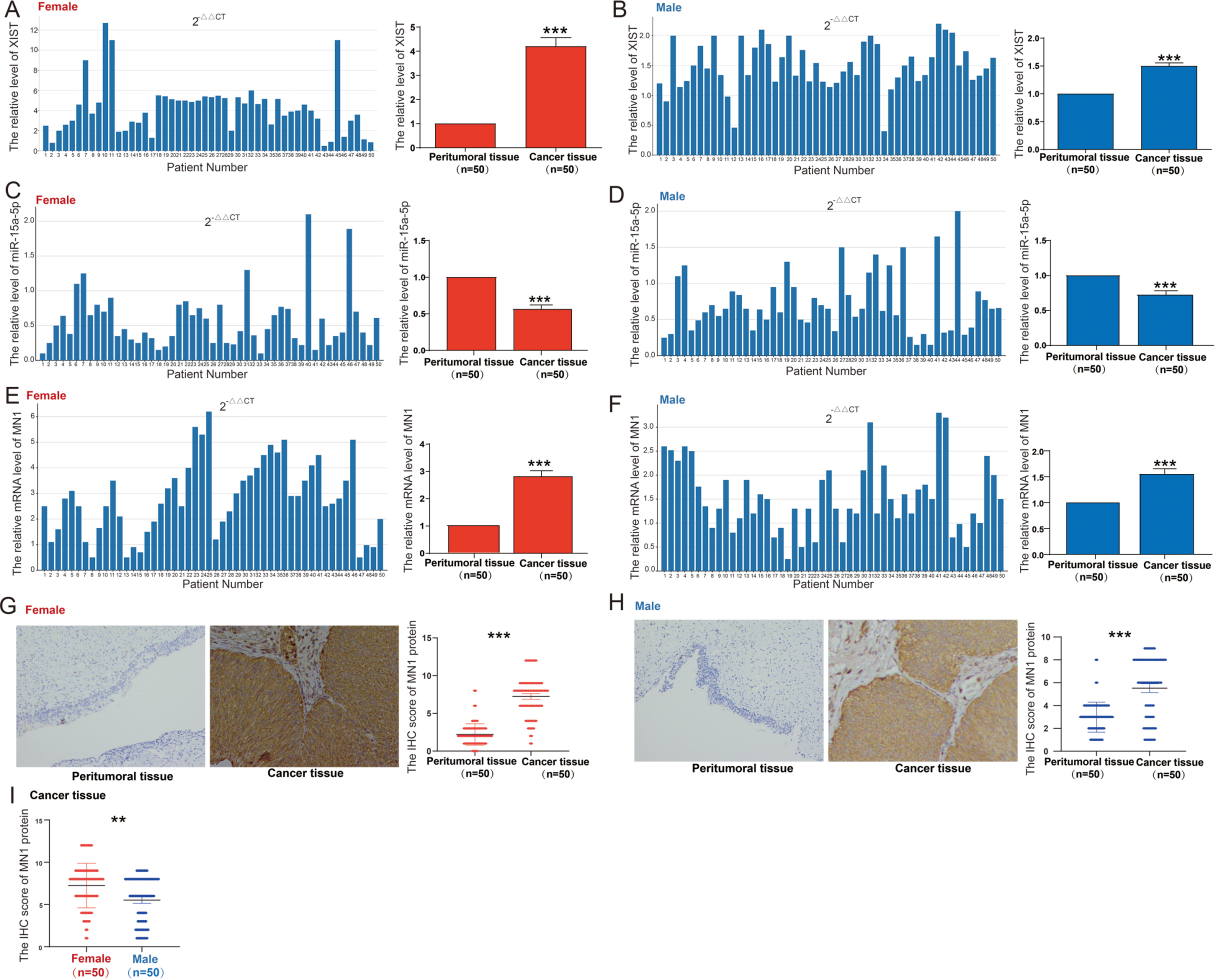
Expression of XIST/miR-15a-5p/MN1 in bladder cancer. **(A, B)** XIST expression levels in **(A)** 50 female and **(B)** 50 male bladder cancer patient specimens. **(C, D)** miR-15a-5p expression levels in **(C)** 50 female and **(D)** 50 male bladder cancer patient specimens. **(E, F)** MN1 mRNA expression levels in **(E)** 50 female and **(F)** 50 male bladder cancer patient specimens. **(G, H)** Immunohistochemical analysis of MN1 protein expression in **(G)** 50 female and **(H)** 50 male bladder cancer patient specimens. **(I)** Comparative analysis of MN1 protein expression in male and female bladder cancer patient specimens. ***p<0.001. **p<0.01.

We evaluated the expression of the XIST/miR-15a-5p/MN1 axis in tissue samples from 50 male and 50 female bladder cancer patients. Compared to adjacent non-cancerous tissues, XIST expression was significantly upregulated in both female and male bladder cancer tissues, with a more pronounced increase in females (**Figure 6A, B**). Concurrently, miR-15a-5p expression was markedly downregulated in both female and male bladder cancer tissues compared to adjacent non-cancerous tissues, with a greater reduction noted in females (**Figures 6C, D**). Additionally, MN1 mRNA expression was significantly elevated in both female and male bladder cancer tissues relative to adjacent non-cancerous tissues, with a higher increase in females (**Figures 6E, F**). Immunohistochemical analysis further confirmed that MN1 protein levels were significantly elevated in both female and male bladder cancer tissues compared to adjacent non-cancerous tissues (**Figures 6G, H**), with increased MN1 protein levels observed in female bladder cancer tissues compared to male bladder cancer tissues (**Figure 6I**).

### MN1 knockdown reveals FZD2 as a gender-specific prognostic marker in bladder cancer

**Figure 7:**
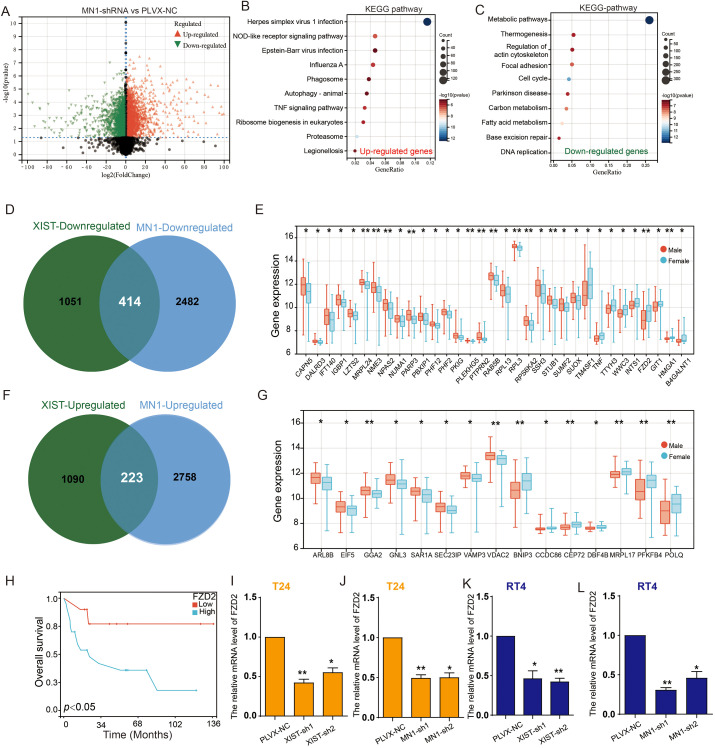
XIST/miR-15a-5p/MN1 regulates FZD2 expression. **(A)** Volcano plot illustrating differentially expressed genes in T24 cells transfected with MN1-shRNA compared to the control group. **(B, C)** KEGG pathway analysis of **(B)** upregulated and **(C)** downregulated genes following MN1 knockdown. **(D)** Venn diagram depicting the intersection of downregulated genes after XIST and MN1 knockdown. **(E)** Differentially expressed downregulated intersecting genes in male versus female bladder cancer. **(F)** Venn diagram showing the intersection of upregulated genes after XIST and MN1 knockdown. **(G)** Differentially expressed upregulated intersecting genes in male versus female bladder cancer specimens. **(H)** Prognostic analysis of FZD2 in female bladder cancer. **(I, J)** Effects of **(I)** XIST knockdown and **(J)** MN1 knockdown on FZD2 expression in T24 cells. **(K, L)** Effects of **(K)** XIST knockdown and **(L)** MN1 knockdown on FZD2 expression in RT4 cells. *p<0.05, **p<0.01.

To further elucidate the molecular mechanisms by which MN1 influences bladder cancer cell functions, we performed RNA sequencing on T24 cells with MN1 knockdown compared to control groups. The results revealed that, in comparison to the control group, 2,981 genes were significantly upregulated, while 2,896 genes were significantly downregulated in the MN1 knockdown group (**Figure 7A**). KEGG enrichment analysis indicated that the upregulated genes were enriched in pathways including the proteasome, NOD-like receptor signaling, and tumor necrosis factor signaling (**Figure 7B**). Conversely, the downregulated genes were enriched in pathways associated with metabolism, fatty acid metabolism, and carbon metabolism (**Figure 7C**). Venn diagram analysis showed that 414 genes were downregulated, and 223 genes were upregulated following the knockdown of both XIST and MN1 (**Figures 7D, E**). Subsequently, we further analyzed the expression of these intersecting genes in BC patients using the GSE13507 cohort. Among the 414 genes downregulated with both XIST and MN1 knockdown, 33 genes exhibited significant differential expression between male and female BC tissues. Notably, TM4SF1, TNF, TTYH3, WWC3, INTS1, FZD2, GIT1, HMGA1, and B4GALNT1 were significantly more highly expressed in female BC tissues compared to male BC tissues (**Figure 7F**). Among the 223 genes upregulated with both XIST and MN1 knockdown, 15 genes showed significant differential expression between male and female BC tissues, with ARL8B, EIF5, GGA2, GNL3, SAR1A, SEC23IP, VAMP3, and VDAC2 being significantly more highly expressed in male BC tissues than in female BC tissues (**Figure 7G**).

Interestingly, survival analysis of these differentially expressed genes indicated that high expression of FZD2 is associated with poor prognosis in female BC patients (**Figure 7H**). Furthermore, knockdown of XIST and MN1 significantly reduced the expression of FZD2 in both T24 and RT4 cells (**Figures 7I–L**).

### XIST/miR-15a-5p/MN1 promotes bladder cancer cell proliferation and metastasis through FZD2

**Figure 8:**
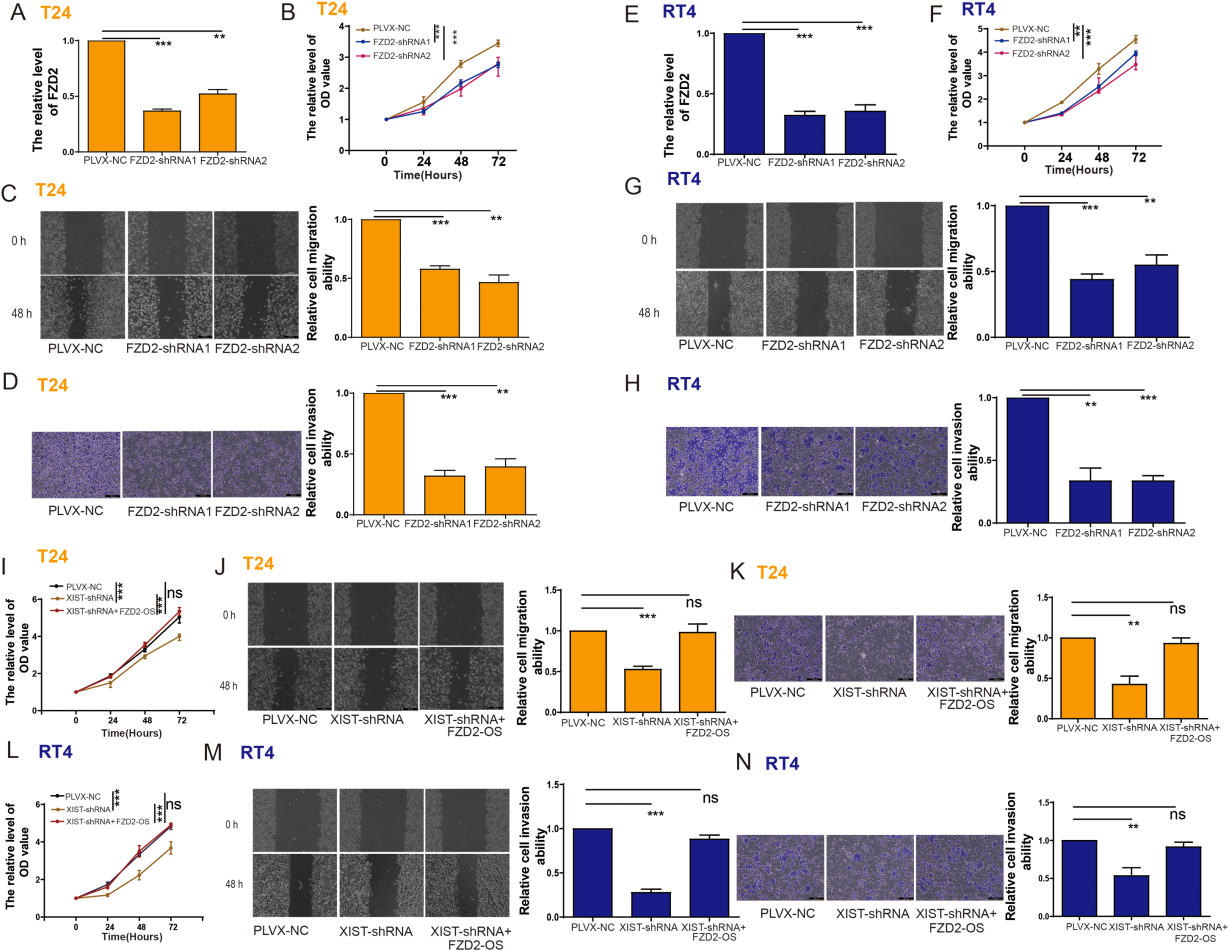
The impact of XIST on the proliferation and metastasis of bladder cancer cells is related to FZD2. **(A)** RT-PCR analysis of FZD2 expression in T24 cells following FZD2 knockdown. **(B–D)** Impact of FZD2 knockdown on the **(B)** proliferation, **(C)** migration, and **(D)** invasion capabilities of T24 cells. **(E)** RT-PCR analysis of FZD2 expression in T24 cells following FZD2 knockdown. **(F–H)** Impact of FZD2 knockdown on the **(F)** proliferation, **(G)** migration, and **(H)** invasion capabilities of T24 cells. **(I–K)** Impact of XIST-shRNA and FZD2-OS transfection on the **(I)** proliferation, **(J)** migration, and **(K)** invasion capabilities of T24 cells. (**L–N)** Impact of XIST-shRNA and FZD2-OS transfection on the **(L)** proliferation, **(M)** migration, and **(N)** invasion capabilities of RT4 cells. ns, p>0.05. **p<0.01, ***p<0.001.

To assess the possible role of FZD2 in bladder cancer cell functions, we knocked down FZD2 in T24 cells (**Figure 8A**). Functional assays revealed that FZD2 knockdown significantly inhibited T24 cell proliferation, migration, and invasion (**Figures 8B–D**). Similarly, FZD2 knockdown markedly suppressed the proliferation, migration, and invasion of RT4 cells (**Figures 8E-H**).

We further investigated whether the effects of XIST on bladder cancer cell functions are mediated through FZD2. Indeed, our findings demonstrated that XIST knockdown significantly reduced the proliferation, migration, and invasion of T24 (**Figures 8I-K**) and RT4 (**Figures 8L, M**) cells. However, these suppressive effects were reversed by FZD2 overexpression (**Figures 8N, O**).

## Discussion

This study explored gender differences in the prognosis of BC and the underlying molecular mechanisms. We identified nine genes with different expressions based on gender in BC, all of which are located on sex chromosomes. Notably, high expression of the XIST gene was associated with poor survival rates in female BC patients, whereas it did not significantly affect male BC patients’ survival. The other eight genes on the Y chromosome were not significantly related to the prognosis of either gender. This suggests that XIST may play a key role in the poorer prognosis observed in female BC patients.

The XIST gene is essential for X chromosome inactivation (XCI) in female mammals, helping to balance gene expression between the sex chromosomes (18). The lncRNA produced by XIST coats the inactive X chromosome and recruits enzymes that modify epigenetic features, leading to changes such as histone deacetylation and DNA methylation (19–21). These changes create transcriptional silencing of the X chromosome, which is crucial for maintaining balanced expression of X-linked genes between the sexes. Although the physiological role of XIST in XCI has been well studied, the effects of its dysregulation on cellular functions need further investigation (22–24).

Previous studies have indicated that dysregulation of XIST is associated with the progression of various cancers (25). For instance, knocking out XIST can inhibit autophagy and carboplatin resistance in ovarian cancer cells through the FOXP1/AKT pathway (26). High levels of XIST enhance the invasiveness and proliferation of prostate cancer cells by sequestering miRNA-96-5p, miRNA-153-3p, and miRNA-182-5p (27). In breast cancer cells, XIST has anti-cancer effects by regulating miR-454 or miR-362-5p, which inhibit cell proliferation and epithelial-mesenchymal transition (EMT), while also inducing apoptosis (28, 29). In bladder cancer, several studies have highlighted the potential role of XIST. For example, Xu et al., found that silencing XIST can inhibit colony formation and EMT in BC cells (30). Bo Hu et al.reported that XIST downregulates p53 expression by binding to TET1, and knocking out XIST inhibits the migration and proliferation of bladder cancer cells (31). Kong et al.reported that XIST promotes bladder cancer progression by modulating miR-129-5p/TNFSF10 axis (32). Additionally, Zhou et al. discovered that XIST promotes BC cell migration and growth by antagonizing miR-133a (33). However, systematic studies examining the specific molecular mechanisms of XIST in relation to gender bias in BC prognosis have not yet been reported.

In this study, we found that XIST might influence bladder cancer progression through the miR-15a-5p/MN1 signaling axis. MN1 is a chromatin remodeling factor and transcription co-regulator that plays a significant role in neural tumors (34). High MN1 expression in gliomas is linked to longer progression-free survival (PFS) (35), while elevated MN1 levels in acute myeloid leukemia (AML) are associated with shorter PFS (36). MN1 also acts as an oncogene in osteosarcoma, where its stability and translation are regulated by specific m6A modifications, contributing to tumor progression and chemotherapy resistance (37). However, the function of MN1 in bladder cancer has not been reported.

Our findings indicate that XIST and MN1 promote the proliferation and migration of bladder cancer cells, while miR-15a-5p exerts inhibitory effects on these processes. Rescue experiments showed that XIST influences the biological functions of bladder cancer cells by regulating MN1 expression. In female BC patient tissues, expression levels of XIST and MN1 were significantly higher, while miR-15a-5p was significantly lower compared to male BC patients. Additionally, we established that the XIST/miR-15a-5p/MN1 axis regulates the expression of the downstream gene FZD2. FZD2 encodes a transmembrane protein with seven transmembrane domains and is involved in regulating several critical signaling pathways. High FZD2 expression is associated with tumor progression in various cancers (38–40).For example, the Wnt5a/FZD2 signaling pathway is critical for resistance to the androgen receptor antagonist enzalutamide in prostate cancer (41, 42); FZD2 activates the Notch signaling pathway to promote TGF-β-induced EMT in breast cancer (43); and WNT2 stabilizes STAT3 signaling through FZD2, modulating metastasis in esophageal cancer. FZD2 regulates limb development by mediating the Wnt/β-catenin pathway (44). The Wnt/β-catenin signaling pathway plays a crucial role in bladder cancer progression. Aberrant activation of this pathway is intimately correlated with heightened bladder cancer invasiveness, increased metastatic potential, and substantially compromised patient prognosis. Mechanistically, this pathway manifests its oncogenic effects through multiple critical processes: stimulating tumor cell proliferation by transcriptionally upregulating survival and growth-associated genes (45); dramatically enhancing cellular migratory and invasive capacities (46); suppressing apoptotic mechanisms; and supporting the maintenance and self-renewal of tumor stem cell populations (47). However, prior reports on FZD2 expression specifically in bladder cancer are lacking. Our data indicate that FZD2 promotes bladder cancer cell proliferation and migration, and that the regulation of these functions by XIST is closely linked to FZD2.

**Figure 9:**
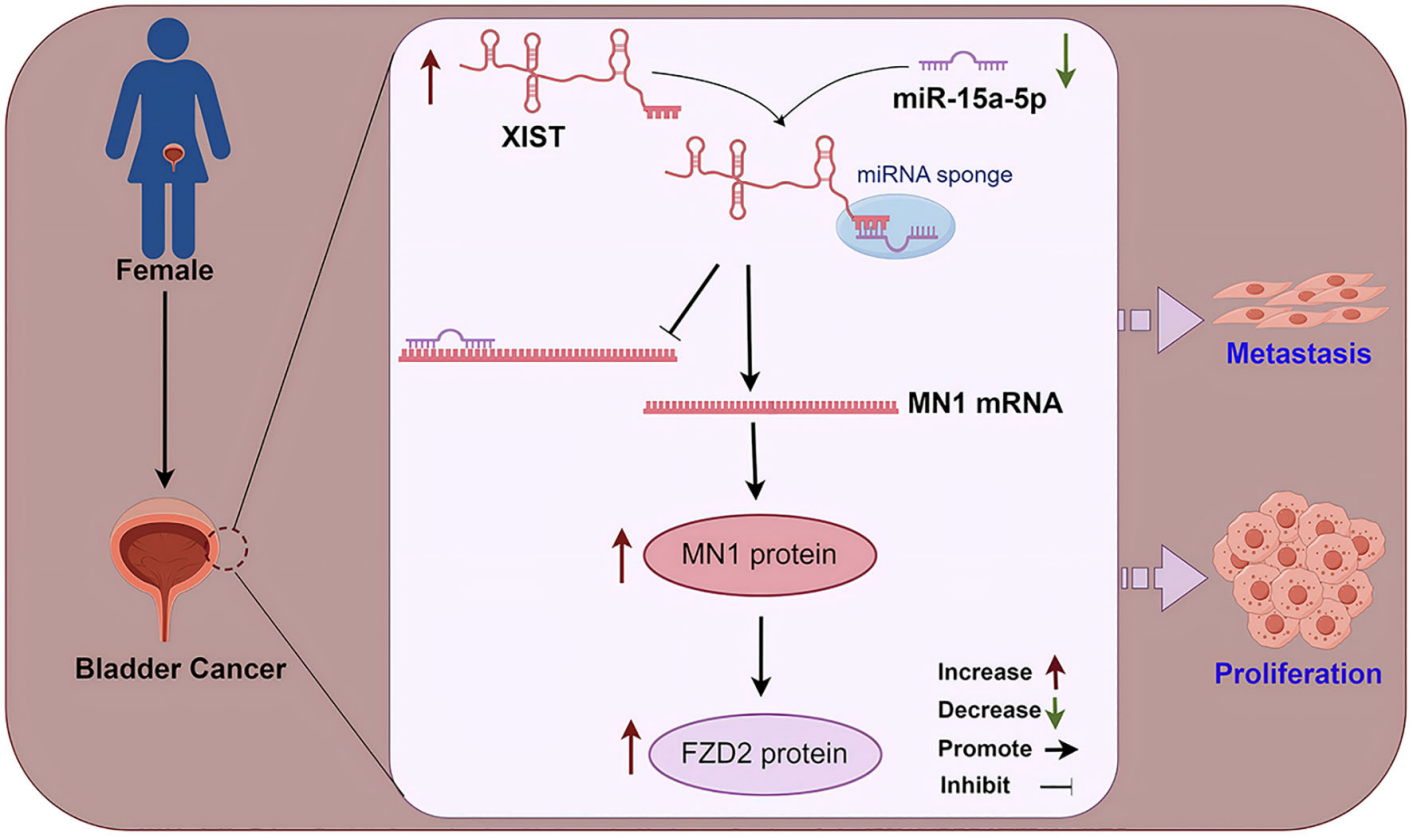
A mechanistic diagram. In female bladder cancer, the XIST/miR-15a-5p/MN1/FZD2 axis is overactivated, thereby promoting the malignant progression of bladder cancer.

In summary, our study explored the molecular mechanisms of the XIST gene in relation to gender differences in BC prognosis. We discovered that the XIST/miR-15a-5p/MN1/FZD2 signaling axis plays a significant role in regulating the progression of bladder cancer (**Figure 9**). This finding provides a new perspective on understanding the gender disparities in bladder cancer prognosis and lays the groundwork for developing new diagnostic biomarkers and therapeutic targets. Nanomaterial-based drug delivery systems (DDS) have emerged as a pivotal approach in contemporary medical and therapeutic domains (48–51). Future research trajectories include leveraging nanomaterials for targeted delivery of therapeutic molecules against XIST and MN1 to bladder cancer cells, with the potential to significantly improve treatment outcomes.

This study has several limitations that warrant further exploration in future research. First, the sample size of 100 surgical specimens (50 males and 50 females), while providing valuable insights, is relatively limited for drawing definitive conclusions about gender-specific differences in bladder cancer. To enhance the robustness and generalizability of our findings, larger-scale multicenter clinical studies with more diverse patient populations are necessary. Such studies would help validate our current observations and provide more comprehensive evidence of the XIST/miR-15a-5p/MN1/FZD2 signaling axis in bladder cancer progression. Additionally, while we propose that FZD2 is a downstream target of MN1, additional experiments, such as chromatin immunoprecipitation assays, are necessary to confirm the direct regulatory interactions between MN1 and FZD2. Establishing this connection would enhance our understanding of the molecular mechanisms underlying the signaling axis and its role in bladder cancer progression. A notable limitation of our current study is the reliance on zebrafish larvae as the primary *in vivo* model. While zebrafish provide valuable insights into cancer mechanisms through real-time fluorescence imaging, they cannot fully recapitulate the complex tumor microenvironment and immunological interactions characteristic of mammalian systems. Rodent models, particularly immunocompromised mouse xenograft models, would offer more translational relevance by providing a more comprehensive assessment of tumor progression, metastasis, and therapeutic responses. Future research should incorporate mouse models to validate our zebrafish findings, investigate long-term tumor development, and explore the potential therapeutic interventions targeting the XIST/miR-15a-5p/MN1 axis in a mammalian context.

## Conclusion

In this study, we identified the critical role of the XIST/miR-15a-5p/MN1/FZD2 signaling axis in explaining gender disparities in bladder cancer prognosis. Our findings show that the upregulation of XIST significantly correlates with poorer prognosis in female bladder cancer patients, pointing to its potential as a novel therapeutic target. By focusing on gender-specific factors, we aim to enhance treatment strategies and clinical outcomes for women diagnosed with this disease.

## Data Availability

The datasets presented in this study can be found in online repositories. The names of the repository/repositories and accession number(s) can be found in the article/supplementary material.
